# Surgical resection as a curative intervention for solitary renal cell carcinoma metastasis to the nasal cavity

**DOI:** 10.1093/jscr/rjae224

**Published:** 2024-04-18

**Authors:** Caroline E Williams, Amir H Sohail, William Smithee, Jose Mercado, Stephen Reynolds

**Affiliations:** General Surgery, UCF/HCA Pensacola, Pensacola, FL 32514, United States; Surgical Oncology, University of New Mexico School of Medicine, Albuquerque, NM 87102, United States; Otolaryngology, HCA Florida Ocala Hospital, Ocala, FL 34471, United States; Otolaryngology, HCA Florida Ocala Hospital, Ocala, FL 34471, United States; Otolaryngology, HCA Florida Ocala Hospital, Ocala, FL 34471, United States

**Keywords:** clear cell renal cell carcinoma, atypical metastasis, nasal cavity metastasis

## Abstract

Persistent unilateral nasal obstruction with recurrent epistaxis in an adult should raise suspicion of malignancy. Renal cell carcinoma accounts for 90% of all renal malignancies but rarely manifests as a nasal mass. We describe a case of clear cell renal cell carcinoma metastasizing to the nasal cavity.

## Introduction

Metastatic neoplasms in the head and neck can be the initial presentation of renal cell carcinoma (RCC) or appear years after primary diagnosis as in our case. Clear cell renal cell carcinoma (ccRCC) manifesting as a nasal mass is very rare [1–3]. RCC accounts for approximately 0.3% of all malignant tumors, 3% of adult malignancies, and 90% of all renal malignancies. It is often discovered in patients 30–60 years of age with a male–female ratio of 1.5:1. The most common subtype is clear ccRCC accounting for almost 85% [2, 4, 5]. Usual sites of metastasis include lungs (75%), regional lymph nodes (65%), bone (40%), liver (40%), and brain (5%). This case report describes a case of ccRCC metastasizing to the nasal cavity, an extremely rare occurrence [4–6].

## Case report

The patient is a 72-year-old male who presented with complaints of nasal obstruction and recurrent, intermittent right-side nosebleeds for 3 months that had become progressively worse in the last 4 weeks prior to presentation. The patient denied antecedent trauma or recent upper respiratory infection. His medical history was significant only for hypertension, and he denied any anticoagulant or platelet use. He further denied fever or purulent secretions. His surgical history was remarkable for right nephrectomy 2017.

**Figure 1:**
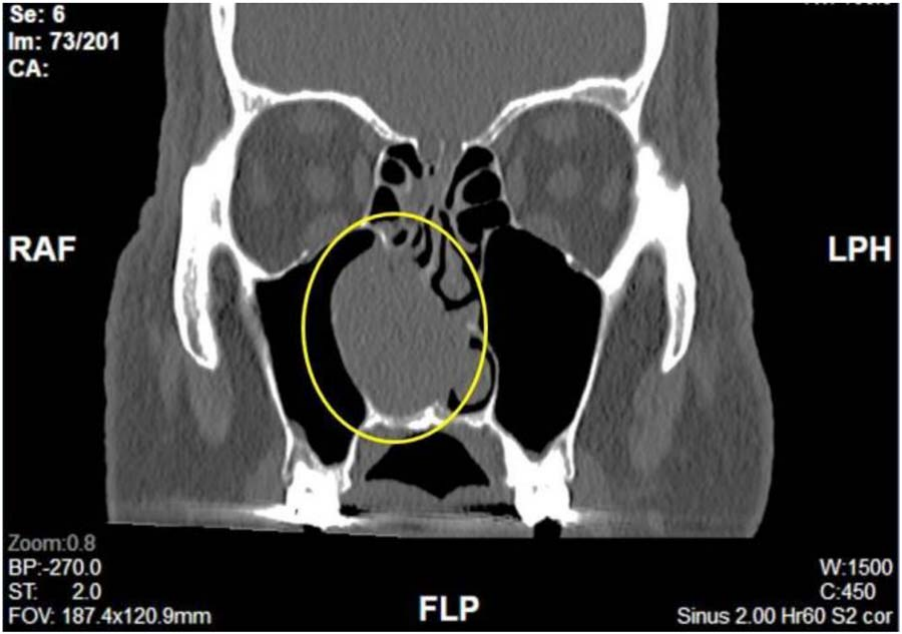
Coronal View of right nasal mass.

**Figure 2:**
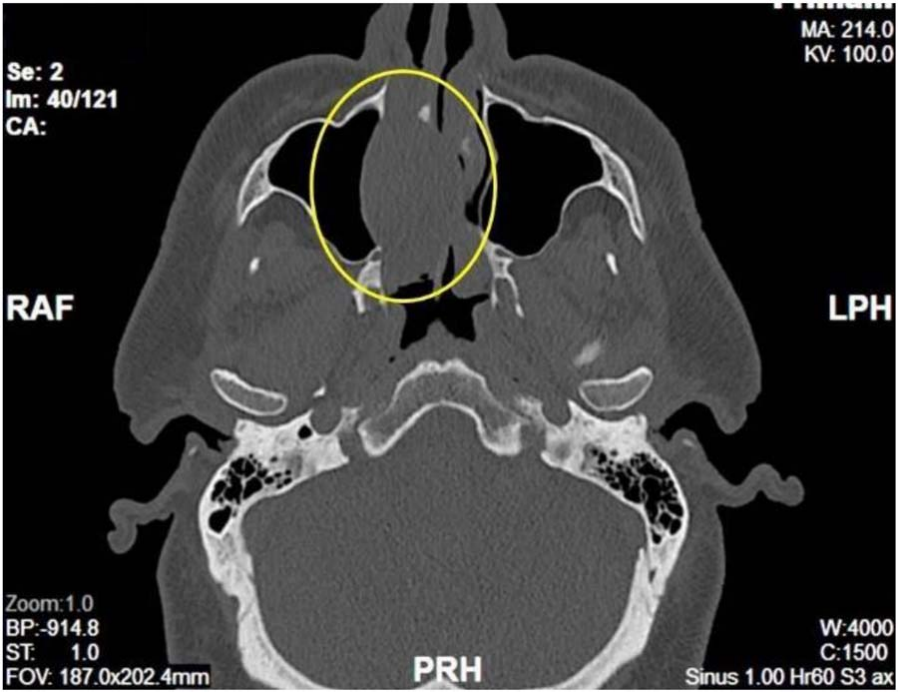
Axial View of right nasal mass.

Complete head and neck examination, including flexible nasal endoscopy, revealed a nasal mass emanating from the right maxillary sinus. There was no active bleeding or purulence on his physical exam. The physical exam of the contralateral side was unremarkable. CT scan of the sinuses with IV contrast revealed an expansile soft tissue mass measuring 4.1 × 2.4 × 3.3 cm. The mass involved the middle turbinate and expanded to the medial wall of the maxillary sinus, blocking the right osteomeatal complex. There was evidence of mucosal thickening of the right ethmoid, right frontal, and right maxillary sinus as well as right septal deviation (Figs 1 and 2). Differential diagnosis based on the physical exam and imaging included inverting papilloma, polypoid mass, vascular lesion, malignant neoplasm, and even an infectious process. The patient was scheduled for functional endoscopic sinus surgery with excision of right nasal cavity mass, right frontal, and maxillary sinuplasty as well as ethmoidectomy and possible septoplasty at the ambulatory surgery center.

**Figure 3:**
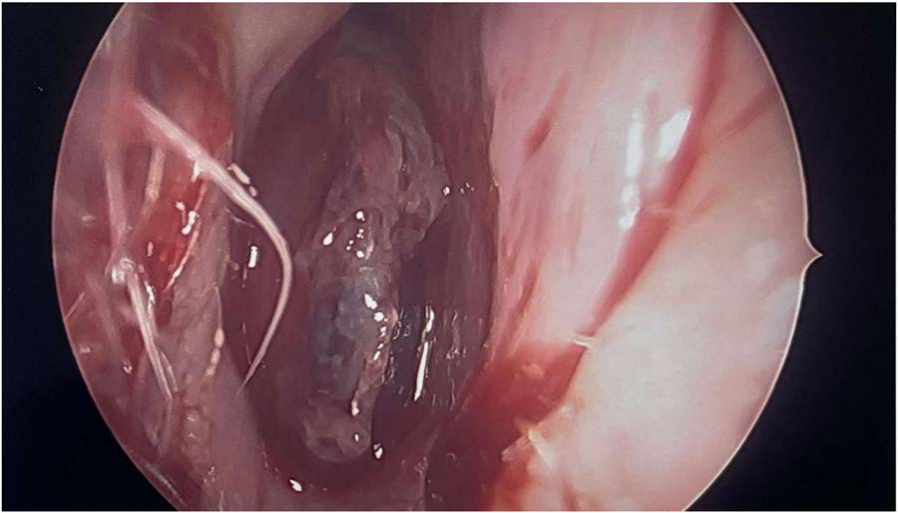
Intraoperative image showing intranasal mass.

Intraoperatively, the right nasal mass appeared hyper vascular (Fig. 3) and bled easily with manipulation. After adequate anesthesia, a zero-degree endoscope and microdebrider were used to take down the right nasal cavity mass. Significant bleeding was encountered during extirpation. The bleeding was successfully stopped via cauterization with suction Bovie and bipolar cautery and topical adrenaline application. Hemostatic packing was placed into the right nasal cavity in order to ensure ongoing hemostasis. At this time, it was determined to no longer be safe to continue the procedure as the patient had lost almost 350 cc of blood and there was concern for recurrent hemorrhage with further manipulation. The decision was made to terminate the procedure and to await biopsy results of the mass prior to further attempts at resection.

**Figure 4:**
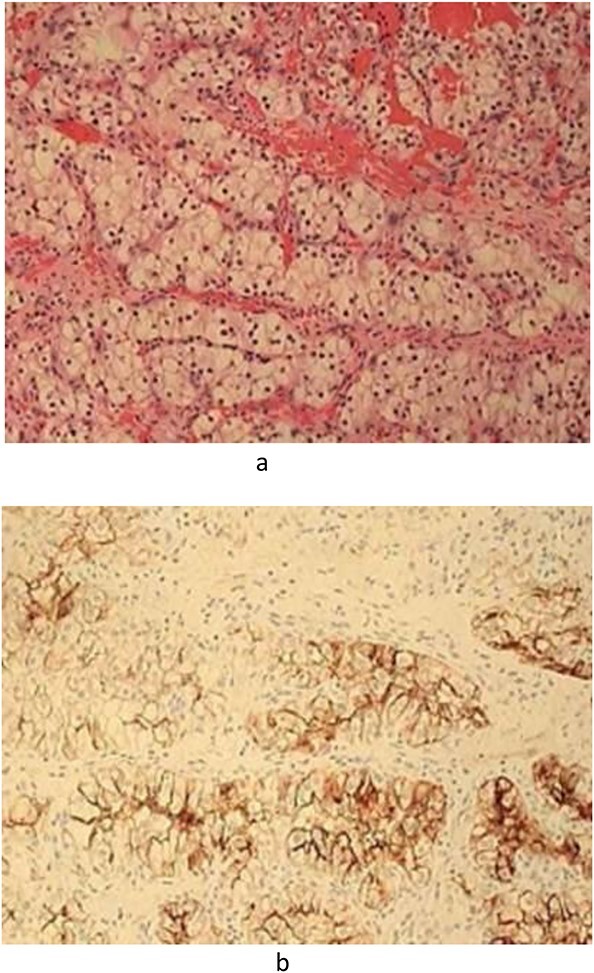
(a) Pathology—Hematoxylin and Eosin-stained section demonstrated fragments of soft tissue with blood representing clear cell malignant population. (b). Pathology—Immunohistochemistry including Cytokeratin AE1/AE3, CD10, RCC, and vimentin were strongly positive.

Postoperatively, histopathologic diagnosis demonstrated bloody fragments of soft tissue along with blood with focal areas representing a clear cell malignant population (Fig. 4a). Immunohistochemistry was performed including cytokeratin AE1/AE3, CK7, CK20, CD10, RCC, S-100, and Vimentin. The clear cell population revealed strong positive staining with cytokeratin AE1/AE3, CD10, RCC, S-100, and Vimentin (Fig. 4b). The findings were morphologically and immunophenotypically consistent with metastatic ccRCC.

## Discussion

In addition to direct visualization, computed tomography with contrast, is the diagnostic modality of choice for nasal manifestations of ccRCC as it highlights the vascular nature of the metastatic tumor. Biopsy to follow can confirm the diagnosis. Treatment options for ccRCC are based on location of primary and metastatic disease as well as overall prognosis. Surgical excision is the primary treatment modality. Intravascular embolization is often recommended prior to surgical excision [1–3]. Local symptomatic control was found in a small group of patients at Christie Hospital in Manchester with a dose of 35 Gy in eight daily fractions [3]. For patients with bone metastasis, disappearance of lesions was noted on bone scan with use of chemotherapy agents such as cabozantinib, a multikinase inhibitor with activity against vascular endothelial growth factor receptors (VEGFR, MET, and AXL) [5].

Multiple new immunotherapy agents have also been implemented in the treatment of metastatic RCC with favorable outcomes [1–3, 7]. The Checkmate 214 trial confirmed the benefit of the nivolumab and ipilimumab association versus sunitinib in first-line metastatic ccRCC among patients with intermediate or poor prognostic risk according to the International Metastatic Database Consortium risk model [8, 9]. Prognosis on ccRCC is often poor [10]. The goal of treatment is to make best use of the existing systemic therapies through personalized approaches so as to improve quality of life. The patient was referred to a tertiary academic center where he ultimately received embolization prior to surgical excision.
